# Analysis of sports records evolution and limits based on integrated features

**DOI:** 10.1038/s41598-024-65350-4

**Published:** 2024-06-24

**Authors:** Lu Tang, Mingliang Yang, Bo Li, Yumin Chen, Yeting Zhang, Xubin Guo

**Affiliations:** 1 Department of Physical Education, Civil Aviation Flight University of China, Guanghan, 618307 China; 2 Integrated Sports Medicine Innovation Hub for Pilots, Civil Aviation Flight, University of China, Guanghan, 618307 China; 3 Institute of Aviation Sports, Civil Aviation Flight University of China, Guanghan, 618307 China

**Keywords:** Evolution, Biochemical networks, Psychology, Human behaviour

## Abstract

Sports records play a crucial role in understanding the limits of human achievement in sports. However, a thorough exploration of a comprehensive analysis of various sports records utilizing the existing statistical model has been lacking. This study introduces a framework for analyzing the integrated features and evolutionary trends of 23 sports records for men and women. It includes world records and intercontinental records from six continents, covering 6440 athletes from 2001 to 2020. Our findings indicate that human beings have not yet reached sports limits in athletic performance, suggesting a continuous improvement over time. Furthermore, we have investigated the contributions of our model’s parameters to the integrated features, emphasizing their robustness and convergence in handling data flow and information entropy. Additionally, our model underscores the significance of integrating various sports for ongoing advancement, in line with the Olympic motto “Together,” thereby promoting coordinated development.

## Introduction

The concept of “sports records” was initially introduced in 1925^1^, and numerous athletes have consistently pursued record-breaking achievements. Furthermore, advancements in population growth, the Industrial Revolution, pharmacology, and the globalization of science and technology have significantly contributed to the progressive documentation of maximum physiological performance in sports records since the inaugural modern Olympic Games in 1896^2–4^. Nevertheless, recent years have seen a regression in sports records, particularly in disciplines such as track and field, attributed to the repercussions of wars and natural disasters^5,6^. There appears to be a plateau in sports records, as suggested by the analysis of current statistical models^2–9^. Interestingly, the new motto of the Tokyo 2020 Olympic Games, ‘Faster, Higher, Stronger-Together^10^, could suggest that only together can we be faster, higher, and stronger. Therefore, this could trigger great and passionate debates about maximum physiological performance in the academic scene across several disciplines^2–9^. However, the statistical models available for the study of a single sports event allow us to remain unclear as to whether the limits of human movement have been reached^4,6^. Therefore, how to interpret sports records and explain the true meaning of the new Olympic motto is a crucial scientific issue^10^. To this end, we will introduce the geometric mean (GM), quantitative difference (QD), I Flow model (IFM), and Information entropy methods to evaluate the development trend of sports records with a comprehensive method instead of a separate method.

Sports records are achieved by different individuals, most likely at different times and locations^11^. Consequently, there are significant variations in the evaluation criteria across various sports disciplines, such as track and field, and swimming, among others. In the context of sports, a complex system refers to the intricate web of interactions and interdependencies between various sporting activities, organizations, and individuals. While each sport may seem independent, they are interconnected through shared resources, as well as through the influence they have on each other and society as a whole^11^. Understanding the emergence of such complexity is challenging due to the intricate interplay between non-linearity, heterogeneity, scale, trade-offs, and synergies^12^. Fortunately, integration serves as a prevalent approach for studying complex systems^13–15^. However, a noteworthy gap remains, as there is a suitable system model capable of simultaneously encompassing temporal and spatial scales to analyze sports records. Therefore, our research aims to introduce a novel perspective that employs quantitative integrated methods to comprehend the complexities inherent in diverse sports records.

Our quantitative integrated approach originates from the Weber-Fechner law, rooted in the foundational principles of psychophysics^16^. This method rigorously combines multiple parameters using the GM^17,18^ to discern the evolutionary trends within sports records. Adhering to the normalization techniques in physical science^19^, we initially derive the IFM through a double normalization process utilizing the GM. The symbol ‘I’ drawn from the Chinese I-Ching, symbolizes the dynamic change of parameters^20^. We then employ QD^21–23^ and information entropy^24^ to define the parameter holes and structure entropy within the IFM. The study hypothesizes that there are universal patterns and constraints governing the limits of athletic performance across diverse sports within the complex system. By analyzing a wide range of sports records quantitatively, the research aims to uncover underlying evolutionary laws that dictate the limits of human potential in athletic performance.

## Methods

### Data source

This study utilized data obtained from the World Athletics (WA) (www.worldathletics.org) and the International Swimming Federation (Fédération Internationale de Natation, FINA) (www.fina.org) to compile comprehensive individual sports datasets for both males and females. To ensure a comprehensive representation across genders in sports, we curated world records spanning six continents (Europe, Central and North America, Asia, Oceania, South America, and Africa) for the time frame spanning 2001–2020.

The inclusion and exclusion criteria for the data adhered to three primary guidelines: 1. Selection of representative Olympic sports; 2. Consistency in standards for both males and females; 3. Limitations of the WA’s official website restricted data availability from 2001 onwards, resulting in a dataset spanning the last two decades. In total, 6440 athletes were considered in the study. The study conducted an analysis covering 23 distinct individual sports events, including 8 track events (100 m, 200 m, 400 m, 800 m, 3000 m, 10000 m, marathon, and 400 m hurdles), 3 field events (high jump, long jump, and triple jump), and 12 swimming events (100 m backstroke, 200 m backstroke, 100 m breaststroke, 200 m breaststroke, 100 m butterfly, 200 m butterfly, 50 m freestyle, 100 m freestyle, 200 m freestyle, 400 m freestyle, 200 m individual medley, and 400 m individual medley).

### Modeling framework

**Figure 1:**
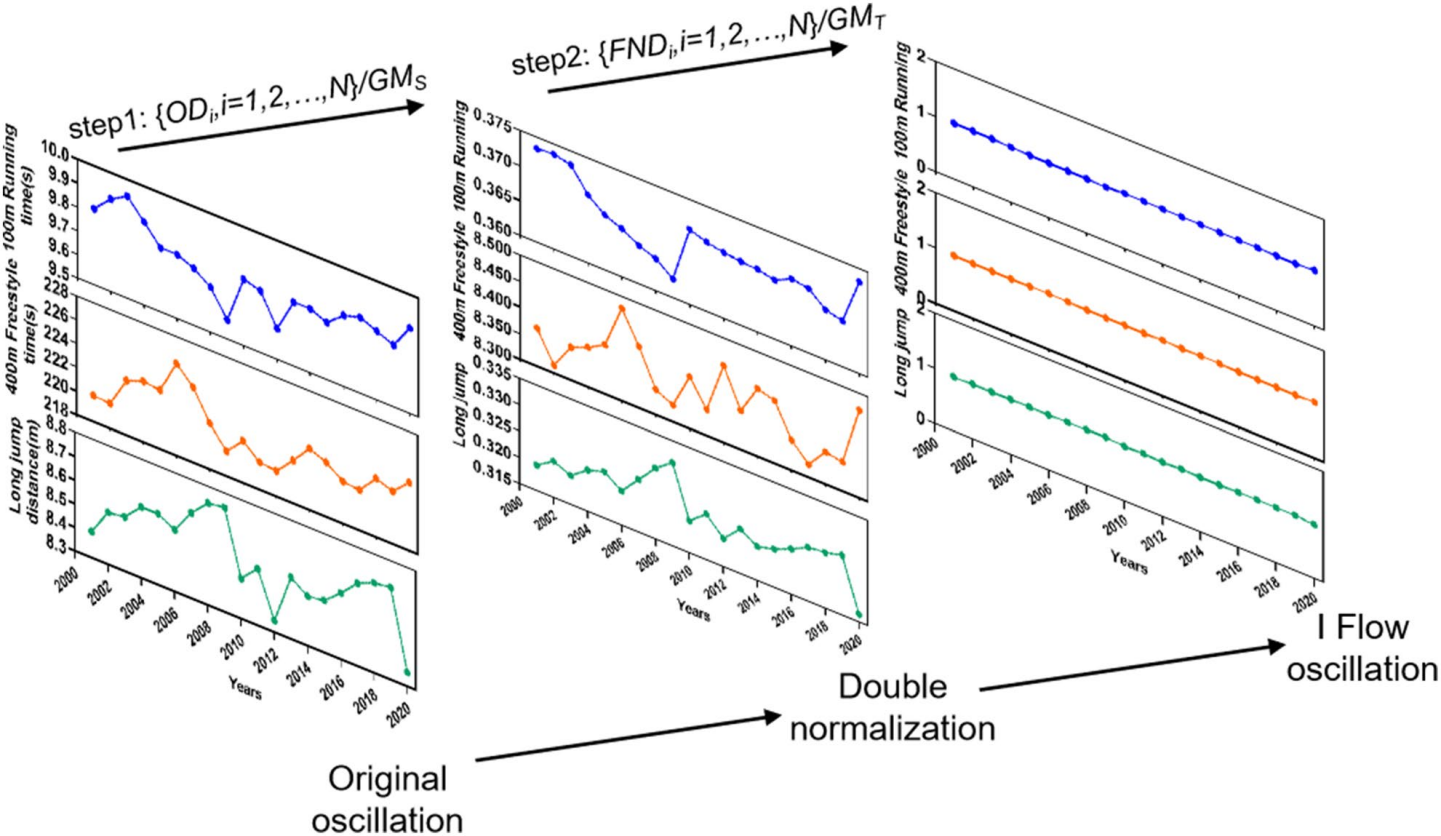
Construction of the I Flow model. As a demonstration, men’s world records in the 100 m running, 400 m freestyle, and long jump have been chosen due to their varying movement distances and action modes. The I Flow oscillation data for these three men’s world records has been converted into a dimensionless format from its original oscillation, consistently maintaining a value around 1 through the application of IFM.

Measuring the central tendency of a system commonly employs means like the arithmetic and geometric means for simplicity. Recent research increasingly favors the geometric mean for its robustness across various studies^25^. Yet, complexities emerge in heterogeneous complex systems due to dimensional inconsistencies among parameters. To address this challenge of comparing parameters with differing dimensions uniformly, we devised a double normalization model. The first step: Divide each parameter of the original data (*OD*) of each sport record $\left\{ {OD_{i} ,i = 1,2, \ldots ,N} \right\}$ by the GM of structure (*GM*_*S*_) to obtain the first normalized data (*FND*) oscillation. The second step: Divide each parameter of the first normalized data of each sports record $\left\{ {FND_{i} ,i = 1,2, \ldots ,N} \right\}$ by the GM of time series (*GM*_*T*_) to obtain the second normalized data oscillation. After double normalization, an integration model of multidimensional data flow will be acquired and defined as IFM, as depicted in Fig. 1. Next, the 23 world records of men and women will be collected to construct IFM.

### Statistical analysis

The GraphPad Prism 8.3 software (GraphPad Software, Inc., San Diego, CA, United States) was used to statistically analyze and visualize the results of the QD value and MED value tests. One-way analysis of variance (ANOVA) and Tukey’s post hoc test were used to compare dependent variables among multiple groups. Data are expressed as mean ± SEM for all tests, with *p*-values of < 0.05 indicating significant difference.

This paper analyzes the quantitative difference (QD)^21–23,26^ of each parameter under the integrated model, instead of analyzing the qualitative difference according to the *P* value. The logarithm based on the golden section, denoted as *τ*, is referred to as the golden logarithm. Within this framework, the QD for a parameter’s two values was established as the absolute value of the golden logarithm derived from their ratio:

1 $$ QD\left( {\frac{{x_{1} }}{{x_{2} }}} \right) = \left| {\log_{\tau } \left( {\frac{{x_{1} }}{{x_{2} }}} \right)} \right|, \tau = \frac{\sqrt 5 - 1}{2} = 0.618 $$

According to Weber–Fechner Law^27^ of psychophysics, through Eq. (1) we have three QD thresholds (*α*,* β*,* γ*), where α called the Weber threshold. If the QD of any two values is less than α, there was not any difference between the two values so the parameter was defined to be biologically conserved. Amdt-Schulz Law^28^ is the dose relationship curve between the classic functions of biomedicine and the influencing factors. *β* and* γ* are defined according to the Amdt–Schulz Law. The QD of any two values in the plateau period of the curve is less than *β*, which was defined as no significant difference, and the QD of any two values in the rising or falling period of the curve is not less than *β* or *γ*, it is defined significant or extraordinary significant difference. The research object of this paper is advanced global body function, that is the *β*_*i*_ threshold of men’s sports records is set as 0.067, while that of women is set as 0.101.

### Information entropy

The Second Law of Thermodynamics dictates that energy gradients invariably disperse, leading to an increase in entropy^29^. An isolated system can either be closed, devoid of energy or matter interaction with its environment, or encompass both the system and its environment if interaction occurs. This law universally asserts that the entropy change in an isolated system is always positive. Shannon’s information entropy^24^ defines a probabilistic distribution law. IFM data, being dimensionless, enables the calculation of information entropy within the IFM parameter space. The hypothesis that the IFM longitudinal space is *N* sports events in a certain year $\left( {x_{1} ,x_{1} , \ldots ,x_{N} } \right)$. Then we sum up the *M* sports events by $\left( {S = \mathop \sum \limits_{i = 1}^{N} x_{i} } \right)$. The *x*_*i*_ parameter is divided by the sum of subspace structures to obtain the probability of subspace structure ${ }\left( {x_{1} /S,x_{1/S} , \ldots ,x_{N} /S} \right)$. Multiply the negative value of the structure probability by the logarithm of the structure probability based on the sum of the subspace structures:

2 $$ H_{i} = - \mathop \sum \limits_{i = 1}^{N} \left( {\frac{{x_{i} }}{S}} \right) \times {\text{log}}\left( {\frac{{x_{i} }}{S}} \right) $$

sum all the parameter of the subspace to obtain the subspace structure entropy:

3 $$ {\text{Entropy}}_{s} = \mathop \sum \limits_{i = 1}^{N} H_{i} $$

Assuming that *x*_*i*_ in Eq. (2) is all 1, then Eq. (3) is the maximum entropy value of 1. To quantify the gap between subspace structure entropy and maximum entropy 1, we derive the maximum entropy distance (MED) as the difference between 1 and subspace structure entropy, termed by $\left( {1 - {\text{Entropy}}_{s} } \right)$. Lower MED values suggest closer proximity to the ideal subspace, coupled with higher maximum entropy.

## Results

### Integrated evolution of sports records

**Figure 2:**
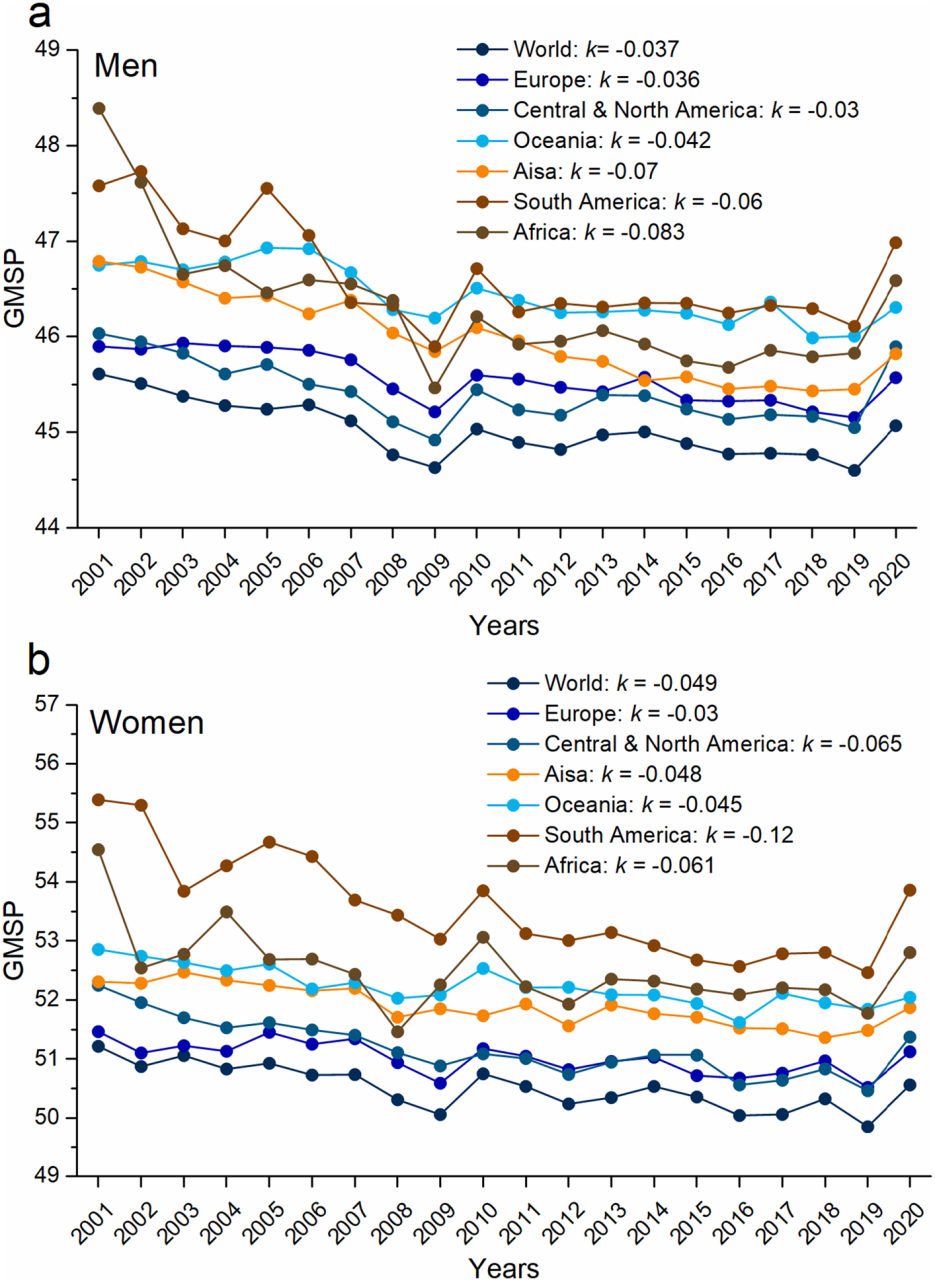
The evolution trend of the geometric mean of sports performance. (**a**) Men. (**b**) Women. Where ***k*** for the slope of polyline.

**Figure 3:**
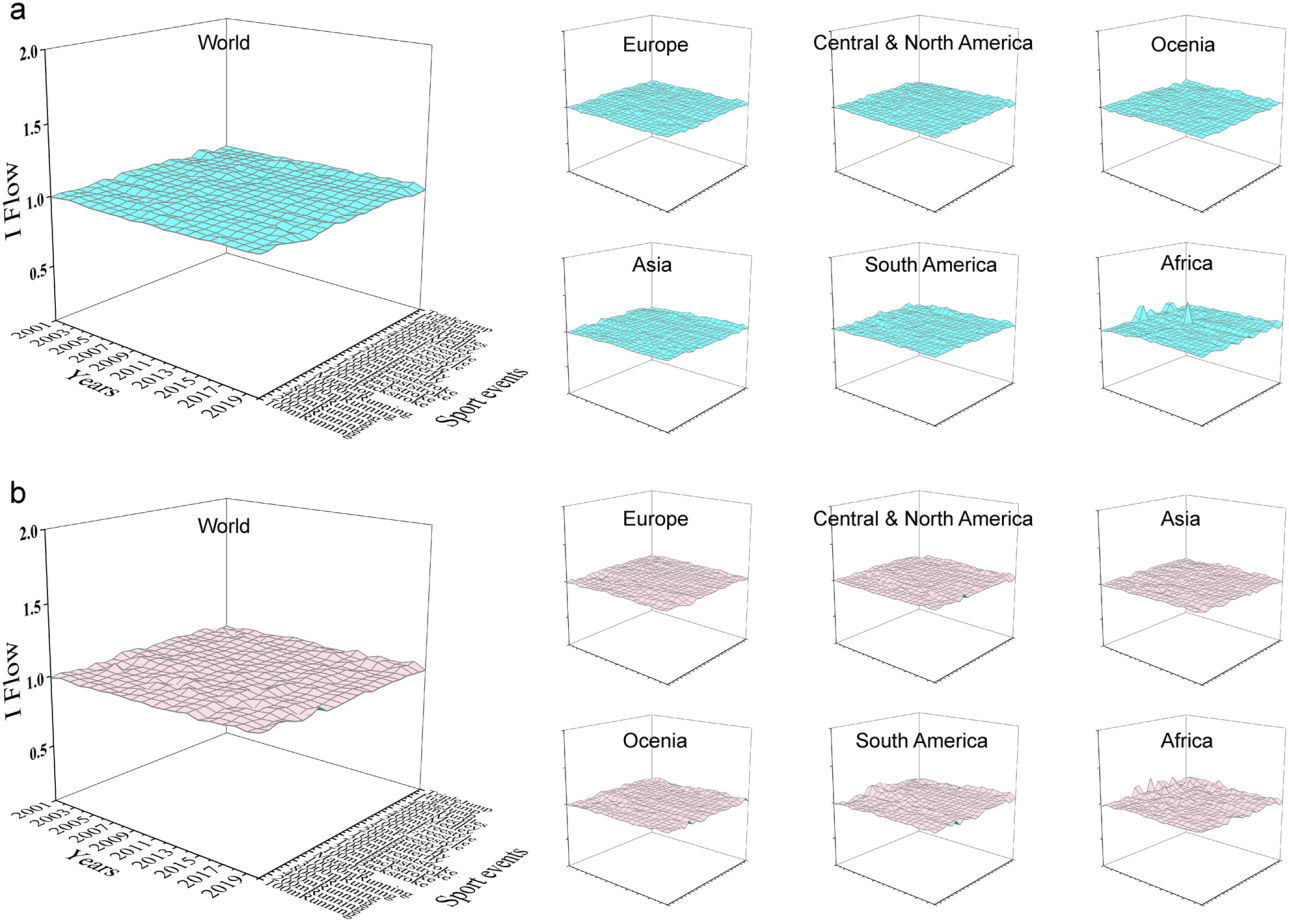
The I Flow model of sports performance. (**a**) Men. (**b**) Women. For each dataset, we show the changes in I Flow data of 23 sports from 2001 to 2020, of which the IF data is dimensionless.

The analysis integrated assessed 23 individual sports events for men and women across six continents using the geometric mean to evaluate Sports Performance (GMSP). Distance functioned as a positive factor in field event evaluation, while time served as a negative factor in track events and swimming. To address this discrepancy, GMSP was computed by reciprocating field event records. Figure 3 illustrates that lower GMSP values indicate superior sports performance (Fig. 2). Notably, world records for both men and women achieve the highest GMSP, while South America and Africa exhibit relatively weaker GMSP. Furthermore, the occurrences of H1N1 influenza in 2009^30^ and the COVID-19 pandemic in 2019^31^ led to a regression in GMSP in 2010 and 2020. Despite this, the consistently negative slope (*k*) in the overall trend suggests an improvement in GMSP and sports performance.

### I flow model of sports records

We utilized the IFM framework to analyze annual sports records, illustrating the relevance of sports events. Figure 6 displays 23 sports records transformed into dimensionless values via IFM. Men’s and women’s I Flow data from the World, Europe, Central & North America hover close to 1. Unexpectedly, South America and Africa exhibit significant fluctuations in I Flow data, deviating considerably from 1 (Fig. 3). This integration of multi-dimensional sports parameter space prompts us to unveil the parameter space’s significance to SP through QD.

### Quantitative difference in the I flow model

**Figure 4:**
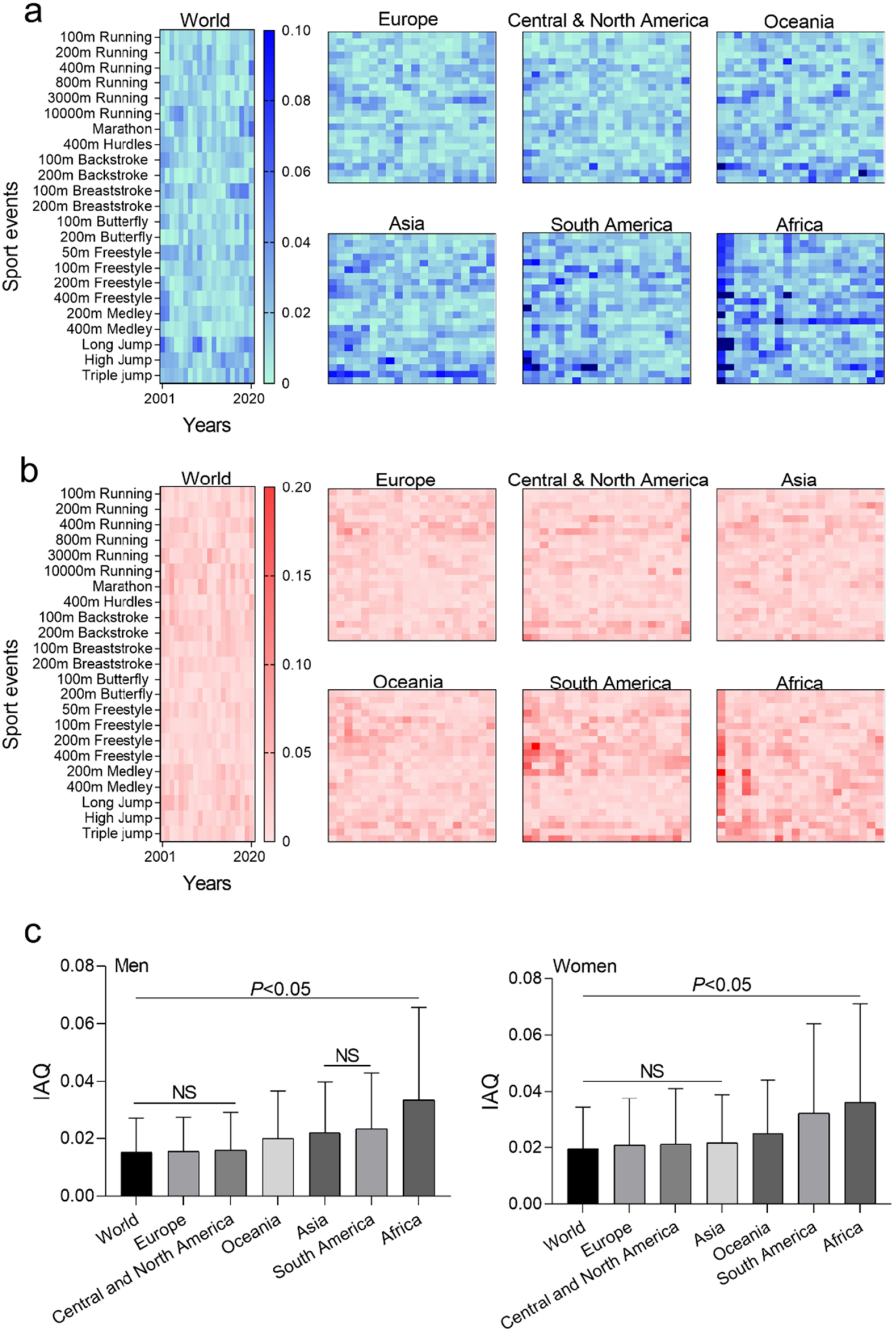
Quantitative differences in the IFM of sports performance. (**a**) Men. (**b**) Women. Each point of the heat map represents the QD between a certain parameter of IFM and 1, in which the darker the color, the larger the QD. If QD > 0.067 is considered as parameter holes for men in a. If QD > 0.101 is considered as parameter holes for women in b. c according to each parameter QD in the heat map, IAQ of the world and six continents is calculated, and the smaller the value, the stronger the correlation of different sports is. Significance calculated using one-way analysis of variance test, followed by post hoc Tukey calculation, significant difference *P* < 0.05, not significant (NS) *P* > 0.05.

The essence of IFM lies in assessing whether each parameter’s entirety falls below the QD threshold. We utilized the IFM average quantitative difference (IAQ) to gauge the collective impact of all parameters, capturing the emergence of numerous interacting individual behaviors in the entirety. IAQ is computed by finding the QD between each I Flow parameter and 1, then deriving the arithmetic mean. Lower IAQ values signify a stronger correlation within the IFM wholes system. As depicted in Fig. 6a and b, world record parameters show the least QD for men and women, revealing no parameter gaps of significance (men: *QD* < 0.067, women: *QD* < 0.101). Notably, South America (16 parameter holes for men, 20 for women) and Africa (44 parameter holes for men, 58 for women) exhibit more pronounced parameter gaps. Figure 6 highlights the lowest IAQ values in world records for men and women. Concerning men’s IAQ, World, Europe, Central and North America register similar levels (*P* > 0.05), followed by Oceania, Asia, and South America at a slightly lower level (*P* > 0.05), while Africa demonstrates significantly lower IAQ than other continents (*P* < 0.05). For women’s IAQ, World, Europe, Asia, Central and North America display comparable levels (*P* > 0.05), followed by Oceania, South America, and Africa. Overall, IFM utilizes QD to evaluate parameter influence on the whole structure (Fig. 4). Our initial findings suggest that sports performance levels depend on the convergence of the I Flow parameter space.

### Attack model node

**Figure 5:**
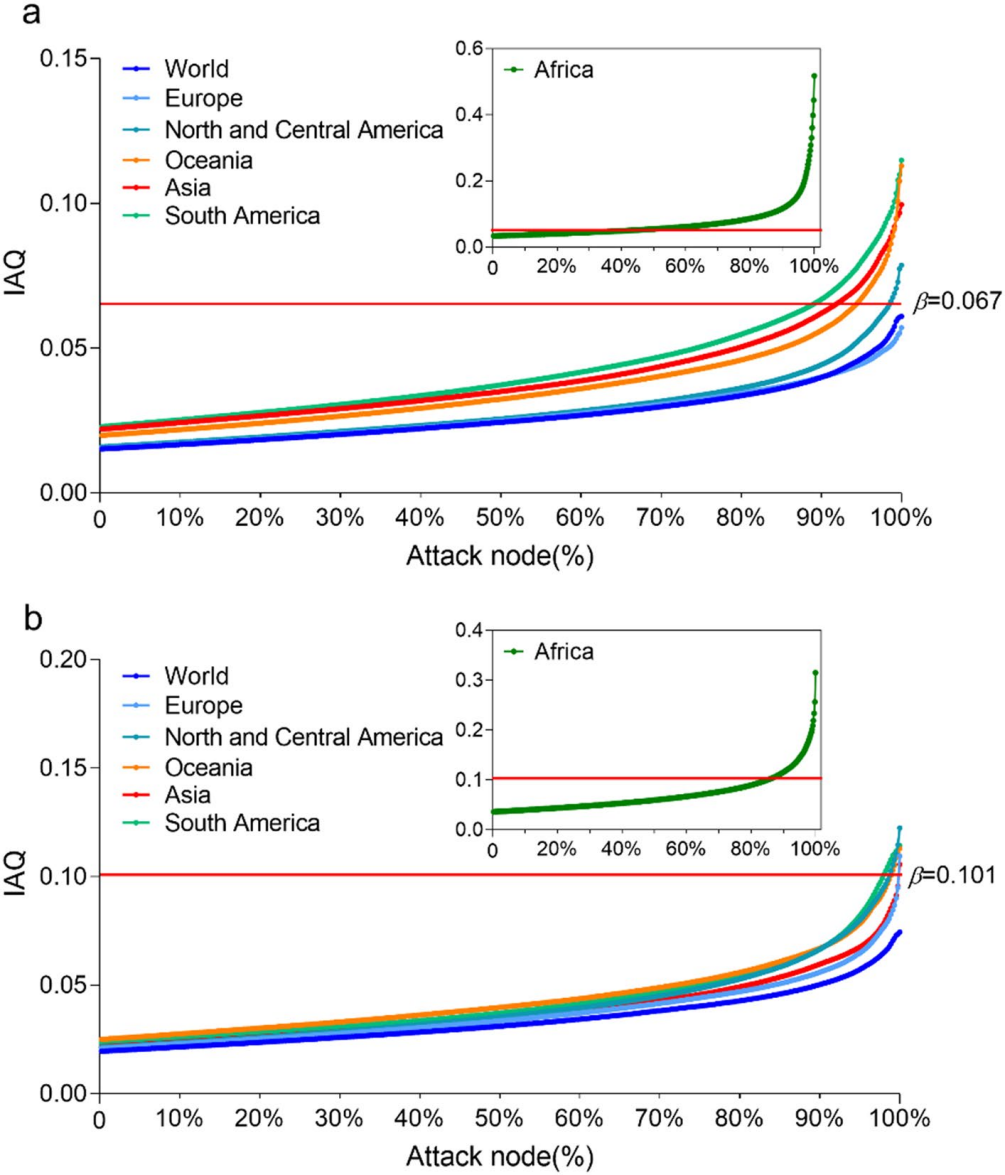
The success rate of the IFM of sports records parameter node from being attacked. **(a)** Men. (**b)** Women. The red horizontal line is the quantitative difference significance threshold (*β* = 0.067 for men, *β* = 0.101 for women).

In complex networks, attacking network nodes to affect node degrees (the connected edge count of nodes) characterizes network robustness and vulnerability^32^. Here, each parameter represents a node, and the node degree is denoted by IAQ. We utilize deliberate attacks (sequentially deleting QD parameters with smaller IFM) and IAQ to test IFM’s robustness and vulnerability. If IAQ surpasses the respective QD threshold, the IFM parameter space is deemed compromised. Figure 5 illustrates the world records’ parameter space is 100% resilient to attacks for men and women. Followed by Europe (100%), Central and North America (98.7%), Oceania (95%), Asia (92.8%), South America (90.2%), and Africa (66.3%) for men (Fig. 5a); and Europe (99.8%), Asia (99.8%), Central and North America (98.7%), Oceania (98.3%), South America (97.8%), and Africa (85%) for women (Fig. 5b). These findings imply that world-class sports events possess substantial anti-interference abilities and robustness within the IFM parameter space, indicating a synchronized and convergent evolution of sports events over 20 years.

### Maximum entropy of integration model

**Figure 6:**
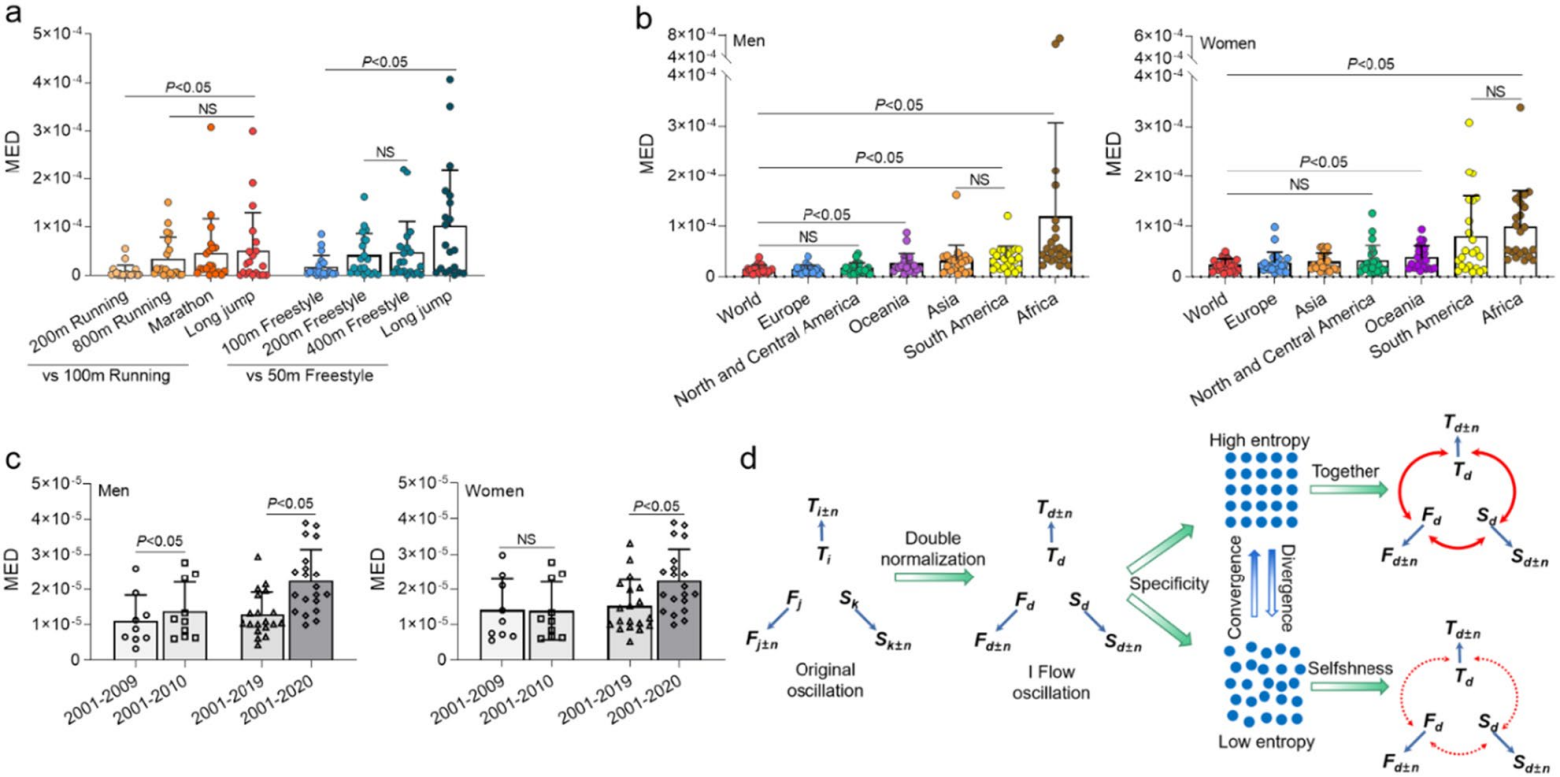
Maximum entropy distance (MED) in the IFM of sports performance. (**a)** Comparison of 100 m running in men’s World records with MED for four sports (200 m running, 800 m running, marathon, long jump), and 50 m swimming with MED for four sports (100 m swimming, 200 m swimming, 400 m swimming, long jump). (**b)** 20-year MED comparison between men and women across the World and six continents. (**c)** Exclusion of the impact of the 2010 and 2020 pandemic viruses on MED. (**d)** Symbols: i for track events records, j for field events records, k for swimming events records, n for sports records evolution; blue arrow denotes the evolution process, and d represents dimensionless data derived from the double normalization of IFM. Thick and dashed red arrows signify together and selfishness, respectively, during the entropy increase evolution process in IFM sports records. Significance determined using one-way analysis of variance followed by post hoc Tukey calculation: significant difference *P* < 0.05, not significant (NS) *P* > 0.05.

The IFM’s maximum entropy state symbolizes parameter space convergence, measured using MED to illustrate the distance between entropy and the maximum value of 1. Figure 6a verifies MED accuracy by comparing men’s world records for 100 m and 200 m running, 800 m running, marathon, and long jump. Similarly, comparisons between 50 m freestyle and other swimming events are made. The results reveal the smallest MED for 100 m and 200 m running, significantly lower than 800 m, marathon, and long jump (*P* < 0.01). Notably, as running distance increases or physical activity differs from the long jump, MED progressively rises for 100 m, 200 m, 800 m, marathon, and long jump. Similar patterns emerge in swimming events. These findings demonstrate that closer sports distances or similar modes result in smaller MED values, indicating stronger convergence. This confirms the IFM’s maximum entropy state as representative of convergence.

We proceed to analyze and compare the structure (sports events) entropy of IFM across men’s and women’s World records and six continents over 20 years. In Fig. 6b, MED exhibits no significant difference among male athletes in the World, Europe, Central and North America (P > 0.05), but significantly differs from other continents (*P* < 0.05). Notably, Oceania’s MED is significantly lower than other continents (*P* < 0.05), while Asia and South America show no differences (*P* > 0.05), yet lower than Africa (*P* < 0.05). Among women’s sports, MED shows no significant difference between the World, Europe, Asia, Central and North America (*P* > 0.05), but significantly differs from other continents (*P* < 0.05). Notably, Oceania’s MED significantly differs from South America and Africa (*P* < 0.05). Overall, these results highlight converging developments among men’s sports in Europe, Central and North America, and women’s sports across Europe, Central and North America, and Asia at the highest global level. Conversely, sports events in Africa exhibit notable divergence between men and women, aligning with GMSP and IAQ findings mentioned earlier. These findings contribute to understanding how MED integrates IFM parameter space, influencing interactions within sports events.

Figure 2 shows that GMSP has regressed in 2010 and 2020, we consider that it may be caused by the H1N1 Virus^35^ in 2009 and COVID-19 in 2019^36^. Assuming the removal of virus-related factors impacts MED, Fig. 6c depicts a significant drop in MED for global men’s and women’s records when excluding data post-2020 (calculating MED solely for 2001–2019), with men’s MED notably dropping post-2009 (calculating MED solely for 2001–2009). This highlights the pandemic virus as the primary interference in sports performance. Fortunately, the impact of the first turning point (2010) on sports performance over the subsequent decade suggests the ‘virus’ effect is temporary, not impeding the trend of improving sports records.

These results suggest that transitioning from a parameter hole to a parameter-free state signifies an increase in information entropy. Entropy rise leads to parameter space convergence, while entropy reduction results in parameter space divergence (Fig. 6d).

## Discussion

This article aimed to introduce a new method for analyzing integrated features of 23 sports world records for men and women across six continents from 2001 to 2020. Key findings include: (1) Smaller GMSP values indicate higher SP by quantifying parameter centrality using geometric mean; (2) Sports records exhibit a trend of continuous improvement, suggesting human sports records have not reached their limits; (3) The structure entropy aligns with the Second Law of Thermodynamics.

Complex systems pose challenges for evaluation using available mathematical models. The geometric mean displayed robust overall performance, corroborated by available evidence. Due to unit distinctions—distance in field events and time in track events/swimming—smaller GMSP values indicate better sports performance. The negative slope in the GMSP scatter chart over the last 20 years signifies progress towards better records, contradicting previous single sports record-based trend assessments^2–9^. Regarding sports performance, there is no gender disparity; men and women exhibit similar SP levels, and world records approach sports limits. In contrast, African records display the poorest integration characteristics, necessitating further exploration of emerging patterns between segments. Notably, our model indicates GMSP regression in 2010 and 2020, potentially linked to the occurrences of H1N1 Virus^30^ and COVID-19^31^, respectively.

Our model yields insights into how various states and sports impact structural interactions. We apply double normalization, a common method in mathematics and physics^33^, but seldom used in multi-parameter systems. This approach facilitates the comparison of parameters with different dimensions on a common platform, elucidating potential correlations between sports events. The annual world record not only signifies the pinnacle of human sports ability but also reflects the highest sports training level globally. In essence, world records’ integration characteristics aptly mirror humanity’s top physiological state and training wisdom across diverse events.

QD significantly influences characterizing IFM parameter space interactions. Smaller QD between different movements correlates with reduced system parameter holes and improved overall movement performance. The world record IFM parameter space, displays the smallest QD (no parameter holes), and from the robustness and fragility of IFM parameter space, it is found that the world record IFM parameter space exhibits superior anti-interference robustness. In summary, the annual world record reflects the adoption of the most advanced training theories, emphasizing universal laws applicable to all research or sports projects. IFM embodies the equality of diverse sports, promoting collective efforts for overall development on a shared platform. To provide an insightful interpretation of model results, empirical assumptions are necessary. In this study, we assume the model parameter space distribution follows Shannon’s information entropy^24^. In contrast to the general Second Law of Thermodynamics^34^, which applies to homogeneous or identical particle systems, our focus is on highly heterogeneous biological systems. IFM abstracts biological systems into parameter space, replacing material space^17^. Our findings demonstrate a convergence phenomenon in athletic performance across all levels facilitated by double normalization. Considering this is a large-scale system, hazardous factors (parameter holes) disrupt its isolation^21^. Removal of harmful factors returns the giant system to isolation, naturally increasing information entropy and adhering to the Second Law of Thermodynamics^34^. In essence, transitioning from IFM with parameter holes to a perfect IFM represents an increase in information entropy, aligning with the Second Law of Thermodynamics^34^. Therefore, improved performance in different sports is achievable only when unified. An ancient African proverb^35^ emphasizes this: ‘If you want to go far, go together.’ Our results quantitatively support the Olympic Games added motto ‘together’, underscoring that only collective together makes us ‘faster, higher, and stronger.’

Our study has several limitations. Firstly, our results are derived from group data and do not comprehensively evaluate whether sports limits are attained in a particular sport. Individualized athlete-specific models are necessary to enhance performance optimization through tailored training recommendations^36^. Additionally, our model encounters challenges stemming from non-physiological factors such as environmental conditions, participation in the sport, and doping^11^. Particularly noteworthy is the trend of doping practices becoming more covert and sophisticated, which could potentially impact the progression of sports records in manner that current statistical models may fail to accurately capture^37^. Consequently, future iterations of the model may consider integrating perturbation terms or implementing piecewise fitting to address this limitation. Finally, given recent rule changes that potentially impact athletic performance, such as the modification of starting pole construction in swimming in 2009 or the adjustment of rules on false starts in athletics in 2010, our subsequent analysis delves into the potential ramifications of these changes on athletes’ strategies and performance in elite competition.

## Summary

Sports records are valuable indicators for evaluating sports limits, enabling an indirect analysis of athletic performance evolution based on historical records. This study compiled data spanning the past 20 years, including 23 world records and intercontinental records from six continents, comprising a total of 6440 athletes. This comprehensive analysis provides new insights into the evolution of sports records. The results reveal a consistent positive trend in both global and continental sports records, suggesting a continuous enhancement in athletic performance over time. Additionally, IFM identifies convergence among various sports records, suggesting that coordinated development in sports may be necessary to surpass sports limits (Table S1).

### Supplementary Information

Supplementary Tables.

## Data Availability

Publicly available datasets were analyzed in this study. The raw data supporting the conclusions of this article will be made available by the Corresponding authors, without undue reservation. The datasets generated during analysed during the current study are available in the International Amateur Athletics Federation (IAAF) (www.worldathletics.org) and the International Swimming Federation (Fédération Internationale de Natation Association, FINA) (www.fina.org).
