# Supplementary material for: Analysis of sports records evolution and limits based on integrated features
Source: Sci Rep. 2024 Jun 24;14:14554. doi: 10.1038/s41598-024-65350-4 (PMC11196580; doi:10.1038/s41598-024-65350-4)
Supplement: Supplementary file 1 — Supplementary Tables. [file 41598_2024_65350_MOESM1_ESM.docx]

Supplementary Material

# Supplementary Table

Table 1. Mathematical explanation of the operator in this paper

| Nomenclature | Equation | Connotation |
| --- | --- | --- |
| Geometric mean (GM) | $GM\left( N \right)=\left( \prod_{i=1}^{N} x_{i} \right)^{1/N}$ | Indicates a trend towards concentration of the *N* parameters. |
| Double normalization (DN) | $x_{ij}:\mathrm{the}i^{th}\mathrm{parmeter}\left( M \right), the j^{th}\mathrm{group}\left( N \right)$ $\mathrm{DN}: \left\{ x_{ij}^{''} \right\}\underset{\to}{x_{ij}^{''}/\left( \prod_{i} x_{ij}^{''} \right)^{1/M}}\left\{ x_{ij}^{'} \right\}\underset{\to}{x_{ij}^{'}/\left( \prod_{i} x_{ij}^{'} \right)^{1/M}}\left\{ x_{ij} \right\}$ | Parameters (*i^th^*) and groups (*j^th^*) are normalised simultaneously in the parameter system (*x_ij_*), which removes the scale between parameters and allows parameters to be compared in the same platform. |
| Quantitative difference (QD) | $\mathrm{QD}:\left( \frac{x_{1}}{x_{2}} \right)=\left\vert{log}_{\tau} \left( \frac{x_{1}}{x_{2}} \right) \right\vert, \tau=\frac{\sqrt{5}-1}{2}=0.618$ | The QD for a parameter's two values (*x_1_,x_2_*)was established as the absolute value of the golden logarithm derived from their ratio. |
| Information entropy (*H*) | $H(X)=-\sum_{i=1}^{n} P\left( x_{i} \right)\times log(P\left( x_{i} \right))$ | Measures the uncertainty or randomness associated with a random variable or a probability distribution. |
| Maximum entropy distance (MED) | $MED=(1-H(X))$ | Indicates the distance from the maximum entropy 1, and the smaller the value, the closer to the maximum entropy. |
